# Association Between Nutrition Social Behavior Change Communication and Improved Caregiver Health and Nutrition Knowledge and Practices in Rural Tanzania

**DOI:** 10.3389/fpubh.2022.736666

**Published:** 2022-06-20

**Authors:** Frederick Kobina Ebo Grant, Robert Ackatia-Armah, Haile Selassie Okuku, Rogers Kakuhenzire

**Affiliations:** ^1^International Potato Center, Kampala, Uganda; ^2^International Potato Center, Kigali, Rwanda; ^3^International Potato Center, Dar es Salaam, Tanzania

**Keywords:** behavior change, knowledge, nutrition, Tanzania, practices

## Abstract

**Background:**

Efforts to improve infant and young child feeding practices include the use of nutrition behavior change communication among caregivers of children under 5 years. We assessed the association between monthly participation in community-level nutrition group meetings on caregiver health and nutrition knowledge and practices (KPs).

**Methods:**

Data from a community-based cross-sectional survey conducted in the Eastern and Southern Highland Zones of Tanzania were used. Indices were developed for caregivers' knowledge of nutrition, health and childcare, household (HDD) and young child dietary diversity (CDD), and vitamin A (VA) intakes. The comparison of means and proportions was assessed using Student's *t*-test and the Chi-square test, respectively, between the caregivers participating in nutrition group meetings and non-participants. The impact of the number of nutrition meeting attendance on caregiver KPs scores was examined using multiple regression.

**Results:**

Of 547 caregivers surveyed, 49.7% attended nutrition group meetings and received information on nutrition social behavior change communication (SBCC). Overall, 28% of participating women had a moderate level of nutrition knowledge, 62% had a high level of VA knowledge, and 57% had a high level of health and childcare knowledge. Participation in nutrition group meetings was significantly associated with the health and childcare knowledge score (HKS), HDD and CDD scores, and household and young child VA intake; the magnitude of the associations was greater for caregivers who attended at least four meetings.

**Conclusion:**

The findings emphasize the need for programs that seek to address the issues present in the use of nutrition SBCC at the community level to improve maternal or caregiver KPs and subsequently the nutrition status of infants and young children.

## Introduction

Globally, more than 3 million children under 5 years of age die annually due to undernutrition (1). Recommended optimal practices for infant and young child feeding (IYCF) include exclusive breastfeeding for all children from birth to 6 months of age, followed by the introduction of nutritionally adequate and safe complementary foods with continued breastfeeding until 2 years of age or older (2). Tanzania, as in many sub-Saharan African (SSA) countries, continues to face nutrition and food security challenges. Approximately one-third (34%), 14, 5, 58, and 33% of children under 5 years are stunted, underweight, wasted, anemic, and vitamin A (VA) deficient, respectively (3). Furthermore, suboptimal IYCF is widespread with only 59% of children under 6 months being exclusively breastfed and only 9% of children aged 6–23 months old receiving the WHO-recommended minimum acceptable diet—a proxy for food insecurity (3).

The use of social behavior change communication (SBCC) strategies can help promote improvements in child health and nutrition in addition to addressing local traditions and household dynamics (4–7). It can also help improve individual practices on health and nutrition. Specifically, nutrition BCC can be used as a strategy to change nutrition-related behaviors at the community and household levels. Suboptimal IYCF practices predict young child undernutrition (8). However, optimal feeding practices and improved dietary habits brought about by nutrition BCC can potentially improve better health, especially among infants and young children, as well as pregnant and lactating women (9). To improve IYCF practices, it is important to target the behavior change of primary caregivers of such children (10).

Nutrition education is widely used for a range of population groups as a medium to deliver healthy diet and nutrition information and its impact on the nutritional status and growth of children (11–15). However, in SSA, greater efforts are needed to improve both the quality and uptake of nutrition and health education interventions at the community levels. The curriculum and training models used to train community health workers (CHWs) or volunteers need to be strengthened to include adequate nutrition and healthcare contents to enable improvement in the downstream delivery of such education to mothers and caregivers at the household level (16).

Most studies on the use of BCC strategies to improve IYCF practices have a short duration (6–12 months) of intervention and follow-up as well as small sample sizes (an average of 150 study participants) and therefore have not been adequately powered to assess changes in knowledge and practices (KPs) on optimal IYCF (11–15). Furthermore, they are not able to differentiate knowledge and attitude from practices because they are measured simultaneously with the potential for reverse causality (8). The initiatives known as the Viable Sweetpotato Technologies for Africa (VISTA) (17) and the Scaling up Sweetpotato Through Agriculture and Nutrition (SUSTAIN) (18) were implemented in multiple countries (Tanzania, Kenya, Rwanda, Mozambique, Malawi) of SSA by the International Potato Center (CIP) and partners. In Tanzania, the activities of VISTA were supplemented by SUSTAIN. The VISTA project employed a strong community-based nutrition education and SBCC to promote the incorporation of biofortified orange-fleshed sweetpotato (OFSP) into the diets of children aged 6–59 months. The focus of the SBCC was to address factors that hinder the uptake of recommended IYCF practices at the community and household levels. The objective of this current study was to assess the association of the nutrition education program implemented within the VISTA project on nutrition and health knowledge and IYCF practices in rural Tanzania.

## Materials and Methods

### Study Setting

This community-based cross-sectional survey was conducted between August and September 2017 in all the seven VISTA Tanzania project intervention districts. The project districts were Gairo and Ulanga in the Morogoro region; Mufindi and Iringa districts in the Iringa region; and Wanging'ombe, Chunya, and Mbozi districts in the Mbeya region. The villages in the project intervention districts were enumerated in August 2017 in preparation for sampling the villages and households to be surveyed. In the three regions, farming is the main economic activity. In terms of agro-ecology, the Morogoro region falls in the eastern agro-ecological zone, while Iringa and Mbeya regions are in the Southern Highlands agro-ecological zone. Both agro-ecological zones receive the highest annual rainfall in Tanzania and are homes to major water bodies that influence the eco-climate, while the numerous rivers are used for many small-scale irrigation schemes. Maize, cassava, rice, potato, and sweetpotato are the main staple crops grown. Cattle raring, small ruminants as well as poultry farming are widely practiced in these regions. Sweetpotato is produced mainly for home consumption and is consumed as boiled, roasted, or deep-fried storage roots. However, sweetpotato leaves in Tanzania are also consumed in local diets and are a common green vegetable in the rural and urban markets.

At the beginning of the project in 2015, there were no documented data on the proportion of households consuming OFSP to our knowledge, which would have been very important for our research in the project target district as a benchmark. However, the project baseline survey revealed that only 0.4% of the households had consumed OFSP during the previous 24 h (19). Elsewhere in Tanzania, a study conducted in 2012, in the Lake agro-ecological zone, revealed that about 2% of households consumed OFSP at least one time every week (20). We anticipate, through the implementation of the VISTA Tanzania project in these selected districts in the eastern and southern agro-ecological zones, where the prevalence of vitamin A deficiency (VAD) is high (36%) among children of 6–59 months (3), that it will be more beneficial to achieve a higher (10%) consumption of provitamin-A-rich OFSP during the previous 24 h among the participating households.

### Study Population

The study targeted households with children <5 years old (6–59 months). Caretakers of these children were the primary respondents. These primary caretakers also participated in the monthly nutrition group meetings in the communities that were established and run by the CHWs. These primary caretakers were mostly the biological mother of the children or the grandmother. During each monthly group meeting, CHWs provided improved maternal, infant, and young child nutrition counseling (16). The main communication aid for the CHWs was a desk-sized set of counseling cards containing eight lessons with each page on the chart containing illustrated examples of healthy practices on the front, with the accompanying messages on the back (4–5 key messages per topic). The main lessons were as follows: (1) healthy mothers during pregnancy; (2) healthy eating; (3) VA; (4) biofortification; (5) infant feeding; (6) OFSP benefits; (7) growing OFSP; and (8) creating a kitchen garden and planting fruits. At each community group meeting, the CHW was to conduct lesson #5 and present one additional topic. Crucially, at these meetings, cooking demonstrations that utilized OFSP and other locally available nutritious and VA-rich foods were carried out by the CHWs with the full participation of the group members present. The curriculum and training models that were used to train the CHWs were adopted from our previous study in western Kenya (16) and modified and adapted for this current nutrition-sensitive project in Tanzania.

#### Inclusion Criteria

Eligible participants included women of 17–45 years of age, residing in the study villages and who are either the biological mother or primary caretaker of a child (6–23 months) or who have a confirmed pregnancy (i.e., by a health worker). Lastly, women must have resided in the study villages during the period of intervention from April 2015 to August 2017.

#### Exclusion Criteria

Women who are younger than 17 or older than 45 years of age and who are not in their first or second trimester of pregnancy or the primary caretaker of a child (6–23 months of age) were not eligible. Women who did not reside full-time in the study villages during the period of the intervention were not eligible.

There were no known risks to these populations beyond some possible discomfort due to the need to assess certain targeted behaviors of the intervention (OFSP knowledge, production, diet practices, food consumption, etc.) or inevitable survey procedures. This study was conducted according to the guidelines laid down in the Declaration of Helsinki, and all procedures involving research study participants were approved by the Commission for Science and Technology/COSTECH, Tanzania. Written informed consent was obtained from all subjects.

### Sample Size Estimation

The sample size of the end-of-project survey was based on the same principle as the baseline survey of 2015 to enable a comprehensive and objective comparison of the primary KP outcome of household weekly frequency of OFSP consumption among project participants. This outcome was the change in the proportion of households consuming OFSP at least one time a week from 0.4% at baseline to 10% at endline. Similar assumptions were made on expected proportions of household weekly frequency of OFSP consumption and on expected changes according to the data of surveys conducted in the Lake Zone regions of Tanzania (20) and in western Kenya (21, 22). The sample size calculation was done to allow for comparison of the proportions between endline (10%) and baseline (0.4%) data accounting for the complex cluster survey design effect (DEFF of 1.5) (23).

Based on an alpha error of 5% and power of 90%, the best estimate of sample size for the primary outcome of household weekly frequency of OFSP consumption was 426 for the seven project intervention districts. This sample size was distributed proportionately among the seven project intervention districts using the probability proportional to the size sampling technique (24). This sample size would allow comparisons for OFSP knowledge, growing practices and consumption, and dietary practices among households between the baseline and endline. The sample size was therefore increased to 512 households to cater for a 20% non-response.

### Sampling Procedure

The endline survey was conducted in the seven intervention districts of the VISTA Tanzania project. Each district has unique characteristics, including the potential for expanding OFSP production; however, poor nutrition practices and low family income are common features among all the project target districts. The project intervention districts and households were purposively selected because they all fall within USAID Feed the Future's Zones of Influence besides having been used during the baseline survey. The survey used a multi-stage cluster sampling design to select the study respondents (24). The first stage involved selecting sample points (“clusters”) using “probability proportionate-to-size” cluster sampling based on the list of villages from each of the project intervention districts (24). Thus, 50 villages were randomly selected from the total number of villages in the project intervention districts.

A list of all the households in the selected village that met the VISTA Tanzania project target intervention criterion was compiled with the help of village agriculture extension officers (VAEOs). The geographical reference of all eligible households was recorded and included in the sample frame for random selection of the eventual respondents. During enumeration, at least 30 families were selected in each village by the village executive officer and the VAEO assisted by the district-based agricultural experts. Emphasis was placed on families with children between 6 and 59 months old. In case households that failed this criterion were listed, they were not geo-referenced for the purpose of saving time since they will not form part of the population that would be sampled for the survey. Informed consent was then obtained from all the eligible participants. Among these households, 11 were randomly selected for individual interviews. Thus, 550 households, each represented by a young child's primary caregiver, were identified for enumeration as the main part of the endline study. Here, a household is defined as a person or a group of persons, related or unrelated, who live together and share a common source of food. Note that the study was oversampled (from 512 to 550) to ensure we have enough power to be able to detect potential differences in other outcomes. In each of the selected households, the primary caretakers of the children were the primary respondents.

### Interview Modules

During the listing stage, in each village, a village leader was interviewed to gather information on the village's access to services such as agricultural extension services, market and health services, and information on other agricultural, health, or any community development intervention that might be happening in their community. A standardized, structured, smartphone-based questionnaire was used in which the responses per respondent were directly recorded by trained enumerators.

All the survey tools were prepared by the VISTA staff in collaboration with project implementing partners (IPs), reviewed for accuracy and completeness, translated into Swahili, and pre-tested before administering in the field for data collection. Based on the pre-test results, the questionnaire was accordingly modified and finalized in consultation with the IPs. The questionnaire was divided into modules, with questions in each module intended to capture different information, knowledge, and practices among the target population about sweetpotato in general and OFSP in particular. The modules of the questionnaire were as follows:

- **Module A**: Household Contact Information.
- **Module B**: Household Characteristics. The characteristics of households (number of members and assets), household head (age, education, and employment), mother (age, relationship to household head, marital status, education, employment, and parity), and children (age and sex).
- **Module C**: Household Food Security and Dietary Diversity. Household food security was assessed using the FANTA Household Food Insecurity Assessment Scale (HFIAS), which has been previously validated in this context (25). Dietary diversity of the household and caregivers utilized a questionnaire combining the HKI food frequency module informing on the frequency of VA-rich food consumption (25) and the WHO 24-h recall method that focuses on the dietary diversity and acceptable diet (26).
- **Module D**: Nutrition KPs. Sought the mothers' or caregivers' knowledge of nutrition and VA, including OFSP and other VA-rich foods.
- **Module E**: Agriculture. Sought information on agricultural production, use of agricultural products, and income derived from agriculture, including OFSP and knowledge about sweetpotato agronomy.
- **Module F**: Project Exposure and Uptake: That included access to OFSP vines for planting; attending OFSP field days and demonstrations; and if ever participated in pregnant and breastfeeding mother's club run by the village-based CHWs.

At enrollment for each respondent, data were collected on basic socio-demographic characteristics, such as age, marital status, education, occupation, household size, and composition. Data on agricultural resources and household assets were also collected to provide a context for understanding the overall results of this research.

### Field Methods for Data Collection

There were two teams of fieldworkers during the data collection phase of the survey. Each team comprised 11 enumerators, a team leader among enumerators, and a CIP staff as a supervisor. The team leader had the responsibility for visiting teams in the field, ensuring that households are selected accurately, and adequate survey tools and other logistics are available. The supervisor was also responsible for deciding how to overcome unexpected problems. Each problem encountered and each decision made were recorded and included in the supervision report. At the end of each workday, the team leaders conducted a wrap-up session with the team to discuss any problems encountered during the day and reviewed all questions and tracking forms to ensure accuracy and completeness. After a review of each completed computer-assisted personal interview (CAPI)-entered data, a backup was created before closing the day's work through Bluetooth technology.

The interview of each caretaker of the eligible and selected household (HH) took approximately 50–70 min, and questions were asked in the Kiswahili language. Each interview was conducted at the home of the participant after she/he was reminded of the informed consent that was procured during the household listing exercise. At the end of each day, the team leader with the assistance of the supervisor reviewed the completed CAPI questionnaires and discussed issues and concerns about the day's interviews. The issues were addressed using field notes, and if necessary, interviewers would return to pertinent HHs to correct the errors.

### Variable Specifications

#### Dependent Variables

Six outcome variables that characterize maternal KPs were constructed. These variables were as follows: (1) nutrition knowledge score (NKS), (2) HKS, (3) household dietary diversity score, (4) young child dietary diversity score, (5) caregiver VA intake, and (6) young child VA intake.

NKS and HKS were derived from key variables using equally weighted summative item scores (see Annex 1 in Supplementary Materials for a list of survey items used). Weights were not used to generate knowledge scores as the selected items were relatively homogenous and equally important and, therefore, would not benefit from the added complexity of weighting and would risk incorrect weight assignment to items (27). The NKS ranged from 0 to 14 points, and the HKS ranged from 0 to 13 points.

The dietary diversity score (DDS) and VA intake score, for both caregiver/household and young children, were the primary health practices of interest. The caregiver's and young child's DDS were constructed from a 24-h food recall, adding the number of different food groups out of 12, which were consumed by the household within the last day (28). Specifically, for the household DDS, 13 food groups were included in the index calculation for households: (1) cereals, (2) roots and tubers, (3) vegetables, (4) fruits, (5) meat and poultry, (6) eggs, (7) fish and seafood, (8) pulses, legumes, and nuts, (9) milk and milk products, (10) oils or fats, (11) sugar or honey, (12) bio-fortified foods, and (13) miscellaneous foods (beverages and related foods). The OFSP was categorized as a biofortified food with both high energy and VA content. Each food group was scored as 0 if not consumed during the past 24 h and 1 if consumed during the same period. The dietary diversity index was obtained by summing the scores for the 13 food groups. Therefore, the possible range of the dietary diversity index was from 0 to 13. The household DDSs were grouped into tertiles: with a score of 0–3 categorized as “low;” a score of 4 as “medium;” and a score of 5 and above as “high.” For computation of a young child's (aged 6–59 months) DDS, the food groups used were as follows: (1) grains, roots, and tubers, (2) VA-rich plant foods, (3) other fruits or vegetables, (4) flesh foods (meat, fish, poultry, and seafoods), (5) eggs, (6) pulses, legumes, and nuts, (7) milk and milk products, (8) any oil or fat-fried/cooked food, and (9) any bio-fortified staples. Each food group was scored as 0 if not consumed during the past 24 h and 1 if consumed in the same period. The dietary diversity index was obtained by summing up the scores for the nine food groups. The possible range of the dietary diversity index was from 0 to 9. The child dietary diversity scores were then grouped into tertiles, with a score of 0–2 categorized as “low;” a score of 3 as “medium;” and a score of 4 and above as “high.”

The frequency of VA consumption score was calculated using the Helen Keller International (HKI) food frequency index model to assess the household risk level of VAD (25). This model counts the frequency of how certain foods are eaten over time although it suffers from a failure to capture actual amounts of each food consumed. However, this model was validated against biochemical indicators and can be used to adequately predict whether VAD is a public health problem in the population. A household was considered to be at risk of VAD if the mean frequency of consuming VA from animal sources was 4 days per week or less or the mean frequency of total consumption of animal and plant sources of VA was 6 days per week or less.

The frequency of the VA consumption score was calculated by first summing the number of days during the previous week the child or the caregiver consumed VA-rich food from ananimal source. Then, the number of days the child or caregiver consumed VA-rich food from a plant source was summed and divided by 6. The following formula was used in calculating the index:

Weighted total consumption days (*C*_*w*_) = Total number of days animal sources of Vitamin A consumed (*T*_*VA*_) + Total number of days plant sources of Vitamin A consumed (*T*_*AP*_) divided by 6.

The weighted VA consumption score (*C*) is equal to the total number of days the child or mother consumed VA-rich food items from animal sources plus the adjusted consumption from the plant sources. The following animal and plant sources were included in the estimation of the index.

- Animal sources: eggs with yolk, fresh silverfish (*daga*) with intact liver or dried silverfish (*daga*) with intact liver, liver from any animal or bird (e.g., chicken) or fish, butter, cod liver oil, VA-fortified margarine (Blue Band) or fortified oil, Cerelac (fortified packaged cereal), infant formula (e.g., NAN, etc.), blood added as an ingredient (Mutura), and VA-fortified sugar.
- Plant sources: sweetpotato leaves, all kinds of dark green vegetables, carrots, ripe mango, pumpkin, ripe papaya, and orange- and yellow-fleshed sweetpotato.

The cut-off point for adequate frequency of VA intake was 6 for the weighted consumption score.

#### Independent Variables

Participation in community-level nutrition group meetings was the primary independent variable of interest. We hypothesized *a priori* that primary caregiver attendance and participating in nutrition group club meetings was a key source of nutrition and health knowledge acquisition. Further, based on our review of the literature and our previous study in western Kenya (29), we identified other maternal, household, and community-level factors that can potentially confound the association between nutrition group meeting participation and nutrition and health KPs.

**Figure 1:**
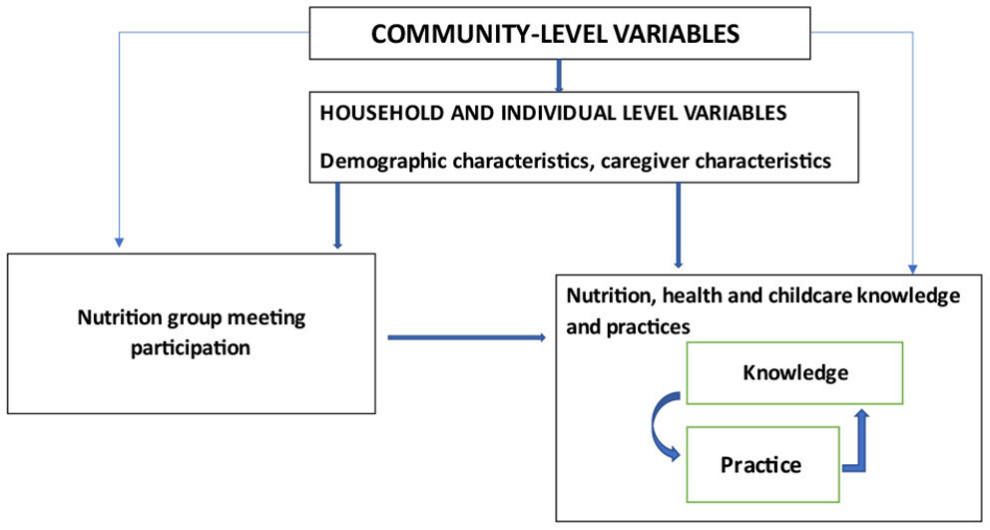
Conceptual framework of the association between participation in nutrition group meetings and knowledge and practices of caregivers of young children. Adopted from Perumal et al. (29).

We used a conceptual framework (Figure 1) adopted from our study in western Kenya (29) to guide the selection of variables for our adjusted regression analysis. At the household level, we considered the status, age, educational level, and employment of the household head, as well as household size as potential confounders of caregiver's participation in nutrition group meetings. Maternal or caregiver socio-demographic characteristics, such as age, marital status, educational status, involvement in agricultural activity and selling agriculture products, engagement in salaried employment, cultivation, and consumption of sweetpotato (OFSP), were identified as potential confounding factors in the association between nutrition social behavior change through club meeting participation and outcome KP variables.

The household wealth index was used as a proxy for the socioeconomic status of the household and was created by summing the values of different predominantly discrete data household variables, such as the type of housing and roofing, the presence and type of toilet, the source of water during the dry season, and the source of cooking fuel, as well as the possession of durable household assets such as radio, television, telephone/mobile, solar panels, gas cooker, bicycle, water pump, motorcycle, car /truck, tractor, and generator. A wealth index based on ordinal variables for these data was created to allow comparison across sites (30). The wealth index scores were then grouped into tertiles, with a score of 0–11 categorized as “poor;” a score of 12–14 as “medium;” and score equal to or >15 as “high.” The maximum score for the wealth index was 29. At the community level, the presence of a nutrition club, village health and nutrition committees, and trained CHWs were considered as factors that potentially affected participation in nutrition group meetings and caregiver KPs.

### Data Management and Analysis

#### Data Management

CSPro-supported CAPI data entry system was used to collect and collate data. In CAPI, the enumerators used smartphones to enter responses on site during the interview. The CAPI application enabled interviews to be conducted face-to-face and determined the question order and performed editing of responses as well as skip patterns. CAPI, therefore, offered a flexible approach to collecting and editing the data, resulted in better data quality, and improved the efficiency of interviewing and final data processing. The endline survey was conducted under a common goal for each village and household sampled in the districts with the intention of pooling the data for analysis. Thus, every effort was made to ensure consistency in the survey execution at every household. All the data were subsequently combined for all the sampled villages and households through a centralized database management system.

#### Data Analyses

After data collection and collation, reports were generated using Stata version 14.1 (StataCorp., College Station, TX) for basic logic, range, and missing data checks. The data were then cleaned and locked for analysis. Descriptive statistics, including frequencies and proportions for categorical variables and mean with standard errors for continuous variables, were generated for study participants.

In bivariate unadjusted analyses, we used Chi-square and Fisher's exact tests (for proportions) and Student's *t-*test (for continuous variables) to compare baseline characteristics of the study participants between the two groups (those attending at least one nutrition group meeting vs. non-attendance). We hypothesized *a priori* that caregivers who participated in nutrition group meetings will demonstrate higher nutrition and health and childcare knowledge and, consequently, better household and young child dietary diversity and VA intake. Differences in nutrition and health and childcare knowledge and dietary diversity and VA intake were compared between the two groups.

With the use of cluster-adjusted regression analysis, we examined the differences between participants and non-participants in the nutrition group meetings. This accounted for the cluster sampling and hierarchical nature of the data. We used multiple linear regression analyses to assess the effect of nutrition group meeting participation (none, <4, and ≥4 visits) on nutrition and health and childcare knowledge, better household and young child dietary diversity, and VA intake, adjusting for caregiver and household-level potential confounders, such as maternal age, marital status, education, employment, household size, sweetpotato cultivation, wealth index, source of consumed OFSP root, and status of head of household. The model was developed by backward stepwise elimination, removing the covariate with the largest *p-*value at each step until the remaining variables were significant at the 0.05 level in the final model. All statistical analyses were assessed by using SAS 9.4 (SAS Institute Inc., Cary, NC). A *p*-value of 0.05 was deemed statistically significant for all analyses. Data are shown as mean ± standard errors (SEs).

## Results

The demographic characteristics of the 547 study participants together with household characteristics are presented in Table 1. Overall, 272 (49.7%) participants indicated to have attended nutrition group meetings and received information on nutrition social behavior change communication (SBCC) with the remaining 50.3% not attending the nutrition group meetings. The mean age of the participating women was 33 years with women who did not attend nutrition group meetings being slightly older (33.6 years) than those who attended (32 years) nutrition meetings. The majority of the women were married (73%), with a significantly greater proportion of married women being in the group that did not attend the nutrition group meetings (76%) compared with those in the group that participated in the nutrition meetings (70%). Women who participated in nutrition club meetings were more likely to have sold agriculture products (43 vs. 24%) and to be involved in the informal business (19 vs. 11%) compared with women who did not participate in the nutrition group meetings. Women who attended nutrition group club meetings were more likely to be in the highest wealth index tertile (47 vs. 35%). Overall, approximately half (47%) of the participants consumed OFSP 24 h prior to the survey, and this did not differ by attendance at nutrition group meetings. Of those who consumed OFSP, more than half (54%) of them obtained the root from their own farms. About 53% of the participants who attended nutrition group meetings sourced their OFSP roots from their own farms compared with 47% of those who did not attend nutrition meetings; however, the difference was not statistically significant (*P* = 0.141).

**Table 1:** Participant and household demographics by whole sample and nutrition group club attendance.

**Variable**	**Overall**	**Club meeting attendance**		***p*-values**
		**Attending**	**Non-attending**	
*n* (%)	547	272 (49.7)	275 (50.3)	
Age of caregiver, years	32.8 ± 0.39	32.1 ± 0.52	33.6 ± 0.57	0.053
**Marital status**, ***n*** **(%)**				
Single	52 (9.5)	24 (8.8)	28 (10.2)	0.006
Married	401 (73.3)	191 (70.2)	210 (76.4)	
Divorce/separated	94 (17.2)	57 (21.0)	37 (13.4)	
**Caregiver/mother education level** ^**a**^				
No schooling	42 (7.8)	21 (7.8)	21 (7.8)	0.459
Primary school completed	428 (79.3)	212 (78.5)	216 (80.0)	
Above primary school	70 (12.9)	37 (13.7)	33 (12.2)	
Household size	4.0 ± 0.07	4.1 ± 0.10	3.9 ± 0.09	0.114
Age of HH head, years	40.0 ± 0.49	39.4 ± 0.66	40.6 ± 0.72	0.227
**Household head**, ***n*** **(%)**^**b**^				
Male headed	463 (84.8)	234 (86.0)	229 (83.6)	0.662
Female with support non-resident male	20 (3.7)	10 (3.7)	10 (3.7)	
Female without male support	63 (11.5)	28 (10.3)	35 (12.7)	
**Household head education level** ^**c**^				
No schooling	30 (5.5)	16 (5.9)	14 (5.2)	0.131
Primary school completed	441 (81.4)	214 (79.3)	227 (83.5)	
Above primary school	71 (13.1)	40 (14.8)	31 (11.3)	
Currently growing sweetpotato, yes	486 (88.9)	241 (88.6)	245 (89.1)	0.856
**Agricultural activity**				
Principal	496 (90.7)	243 (89.3)	253 (92.0)	0.284
Secondary	51 (9.3)	29 (10.7)	22 (8.0)	
Sold agricultural/livestock products in past 2 years, yes	181 (33.1)	116 (42.7)	65 (23.6)	<0.0001
Casual labor in past 2 years, yes	177 (32.4)	80 (29.4)	97 (35.3)	0.143
Involved in informal business within past 2 years, yes	83 (15.2)	52 (19.1)	31 (11.3)	0.011
Salaried employment within past 2 years, yes	46 (8.4)	25 (9.2)	21 (7.6)	0.512
Self-employment within past 2 years, yes	240 (43.9)	124 (45.6)	116 (42.2)	0.422
Wealth index of household	13.65 ± 0.17^1^	14.31 ± 0.23	13.0 ± 0.23	<0.0001
First tertile (lowest)	160 (29.3)	63 (23.2)	97 (35.3)	0.002
Second tertile (medium)	162 (29.6)	80 (29.4)	82 (29.8)	
Third tertile (highest)	225 (41.1)	129 (47.4)	96 (34.9)	
Consumed OFSP within past 24 h, yes	257 (47.0)	125 (48.6)	132 (51.4)	0.243
OFSP from own field	138 (53.7)	73 (52.9)	65 (47.1)	0.141
Market sources	119 (46.3)	52 (43.7)	67 (56.3)	

*^1^ Mean ± standard error (SE)*.

*^a^ Missing values for maternal education, n = 7*.

*^b^ Missing value for the status of household head, n = 1*.

*^c^ Missing values for education status of household head, n=5*.

Unadjusted bivariate comparison in nutrition, VA, HKS, and dietary diversity and dietary VA intake of women and young children by participation in nutrition SBCC are presented in Table 2. Overall, 28% of participating women had a moderate level of nutrition knowledge, 62% had a high level of VA knowledge, and 57% had a high level of health and childcare knowledge; these scores did not differ significantly by nutrition club attendance. Although caregiver DDS did not differ by nutrition group meeting attendance, children of caregivers who participated in nutrition group meetings were more likely to have a moderate level of diet diversity compared with those whose mothers did not participate in nutrition meetings (66.5 vs. 62.6%, *P* = 0.07) as well as a marginally greater number of days of dietary VA intake (5.4 vs. 4.6 days, *P* = 0.053).

**Table 2:** Knowledge and practices of primary caregivers of children under 5 years, overall and by nutrition club meeting attendance.

**Variable**	**Overall**	**Club meeting attendance**		***p*-values^**1**^**
		**Attending**	**Non-attending**	
*n* (%)	547	272 (49.7)	275 (50.3)	
Nutrition knowledge score	7.35 ± 0.13^@^	7.51 ± 0.19^@^	7.19 ± 0.19^@^	0.227
Low (<5)	231 (42.2)	109 (40.1)	122 (44.4)	0.241
Moderate (5–7)	157 (28.7)	75 (27.6)	82 (29.8)	
High (7–14)	159 (29.1)	88 (32.4)	71 (25.8)	
*Vitamin A knowledge score*	3.42 ± 0.06	3.51 ± 0.09	3.33 ± 0.09	0.157
Low (<2)	209 (38.2)	98 (36.0)	111 (40.4)	0.297
High (2–7)	338 (61.8)	174 (64.0)	164 (59.6)	
Health and childcare knowledge score	9.32 ± 0.08	9.36 ± 0.12	9.28 ± 0.11	0.623
Low (<6)	31 (5.7)	13 (4.8)	18 (6.6)	0.525
Moderate (6–9)	203 (37.1)	98 (36.0)	105 (38.2)	
High (10–13)	313 (57.2)	161 (59.2)	152 (55.3)	
Household dietary diversity score	6.66 ± 0.08	6.70 ± 0.11	6.62 ± 0.12	0.640
Low (<4)	62 (11.3)	24 (8.8)	38 (13.8)	0.160
Moderate (4–7)	317 (58.0)	165 (60.7)	152 (55.3)	
High (8–13)	168 (30.7)	83 (30.5)	85 (30.9)	
Young child dietary diversity score	4.61 ± 0.07	4.75 ± 0.09	4.47 ± 0.11	**0.048**
Low (<3)	45 (8.2)	15 (5.5)	30 (10.9)	**0.072**
Moderate (3–5)	353 (64.5)	181 (66.5)	172 (62.6)	
High (6–9)	149 (27.3)	76 (28.0)	73 (26.5)	
Caregiver vitamin A intake	4.93 ± 0.19	5.24 ± 0.31	4.62 ± 0.22	0.101
<6 days	404 (73.9)	198 (72.8)	206 (74.9)	0.574
Above 6 days	143 (26.1)	74 (27.2)	69 (25.1)	
Young child vitamin A intake	5.03 ± 0.20	5.41 ± 0.32	4.65 ± 0.23	**0.053**
<6 days	390 (71.3)	189 (69.5)	201 (73.1)	0.351
Above 6 days	157 (28.7)	83 (30.5)	74 (26.9)	

*^1^ Student's t-test: contrasting mean values between caregivers who participated and those who did not participate in mother club meetings. Pearson's Chi-square test for proportions*.

*^@^ Mean ± standard error (SE) for continuous scale; the scores are categorized for descriptive purposes*.

*The following are the meaning of the bold values: 0.048 (statistically significant at p < 0.05); 0.072 (statistically significant at p < 0.1); 0.053 (statistically significant at p < 0.1)*.

In adjusted analyses, participation in nutrition group meetings was significantly associated with HKS, household and young child DDS, and household and young child VA intake, controlling for maternal age, marital status, education, employment, household size, sweetpotato cultivation, wealth index, and status of head of household (Table 3). Compared with no participation in nutrition group meetings, <4 nutrition meeting attendances were positively associated with greater health and childcare knowledge [β (SE):1.062 (0.187), *P* < 0.001], household [1.300 (0.157), *P* < 0.001] and young child dietary diversity [0.427 (0.127), *P* < 0.01], and household [0.575 (0.317), *P* < 0.01] and young child VA intake [0.827 (0.326), *P* < 0.01]. The magnitude of these associations increased significantly for ≥4 nutrition club meeting attendances {health and childcare knowledge [1.194 (0.248), *P* < 0.0001], household DDS [1.513 (0.209), *P* < 0.0001], young child DDS [0.594 (0.168), *P* < 0.001], household VA intake [1.403 (0.421), *P* < 0.001], and young child VA intake [1.409 (0.433), *P* < 0.001]}. In unadjusted and adjusted analyses, there was no significant difference in nutrition knowledge on participation in nutrition group meetings. Although households who obtained their consumed OFSP from their own farms had better household [0.384 (0.241), *P* = 0.11] and young child [0.316 (0.201), *P* = 0.12] DDS compared with those who bought the root from the market/or obtained from neighbors, there was no statistical difference. Improved wealth index was associated with improved dietary diversity and VA intake for both households and young children.

**Table 3:** Effect of mother club participation on knowledge and practices among caregivers in rural Tanzania, adjusted for covariates.

**Characteristics**	**Nutrition knowledge** ^**§**^		**Health and childcare knowledge** ^**§**^		**HH dietary diversity score** ^**§**^		**Young child dietary diversity score** ^**§**^		**Caregiver vitamin A intake**		**Young child vitamin A intake**	
	**Unadjusted** **β (SE)**	**Adjusted** **β (SE)**	**Unadjusted** **β (SE)**	**Adjusted** **β (SE)**	**Unadjusted** **β (SE)**	**Adjusted** **β (SE)**	**Unadjusted** **β (SE)**	**Adjusted** **β (SE)**	**Unadjusted** **β (SE)**	**Adjusted** **β (SE)**	**Unadjusted** **β (SE)**	**Adjusted** **β (SE)**
Intercept	-	7.468 (1.010)	-	7.044 (0.485)	-	2.966 (0.406)	-	2.394 (0.327)	-	1.437 (0.819)	-	0.621 (0.842)
Caregiver age	−0.005 (0.015)	0.018 (0.017)	0.014 (0.01)*	0.029 (0.009)**	0.004 (0.007)	0.005 (0.007)	−0.002 (0.005)	−0.004 (0.006)	0.002 (0.013)	−0.010 (0.015)	0.005 (0.013)	−0.005 (0.015)
**Number of club meeting attendance**												
None	Reference	Reference	Reference	Reference	Reference	Reference	Reference	Reference	Reference	Reference	Reference	Reference
<4 visits	0.291 (0.278)	0.093 (0.280)	1.338 (0.185)***	1.062 (0.187)***	1.768 (0.160)***	1.300 (0.157)***	0.612 (0.125)***	0.427 (0.127)**	0.916 (0.311)**	0.575 (0.317)*	1.193 (0.322)**	0.827 (0.326)*
≥4 visits	0.368 (0.393)	0.252 (0.380)	1.547 (0.250)***	1.194 (0.248)***	2.068 (0.216)***	1.513 (0.209)***	0.825 (0.169)***	0.594 (0.168)**	1.996 (0.422)***	1.403 (0.421)**	2.028 (0.436)***	1.409 (0.433)**
**Marital status**												
Single	Reference	Reference	Reference	Reference	Reference	Reference	Reference	Reference	Reference	Reference	Reference	Reference
Married	−0.669 (0.462)	−1.289 (0.574)*	−0.132 (0.251)	−0.307 (0.292)	−0.192 (0.226)	−0.413 (0.246)	0.077 (0.167)	−0.061 (0.198)	−0.568 (0.414)	−0.314 (0.495)	−0.326 (0.429)	−0.081 (0.509)
Divorce/Separated	0.132 (0.519)	−0.063 (0.593)	−0.626 (0.279)*	−0.887 (0.290)**	−0.760 (0.251)**	−0.790 (0.244)*	−0.160 (0.185)	−0.132 (0.198)	−0.987 (0.460)*	−0.446 (0.492)	−0.755 (0.477)	−0.184 (0.506)
**Caregiver/mother education level**												
No schooling	Reference	Reference	Reference	Reference	Reference	Reference	Reference	Reference	Reference	Reference	Reference	Reference
Primary school completed	0.604 (0.487)	−0.083 (0.543)	0.724 (0.250)**	0.343 (0.260)	0.690 (0.222)**	0.399 (0.217)	0.460 (0.163)**	0.281 (0.175)	0.510 (0.405)	0.095 (0.438)	0.527 (0.419)	−0.023 (0.450)
Above primary school	0.778 (0.588)	−0.218 (0.679)	1.079 (0.312)***	0.700 (0.340)*	1.270 (0.278)***	0.685 (0.285)*	0.891 (0.204)***	0.387 (0.229)	1.980 (0.507)***	0.717 (0.574)	2.216 (0.525)***	0.804 (0.590)
**Household size**	−0.146 (0.084)	−0.174 (0.095)	−0.101 (0.040)*	−0.114 (0.045)*	0.035 (0.036)	−0.012 (0.038)	0.010 (0.027)	−0.031 (0.030)	0.155 (0.066)*	0.073 (0.076)	0.1226 (0.069)	0.032 (0.078)
**Household head**												
Male headed	Reference	Reference	Reference	Reference	Reference	Reference	Reference	Reference	Reference	Reference	Reference	Reference
Female with support non-resident male	1.817 (0.632)**	1.452 (0.631)*	1.402 (0.471)**	0.926 (0.449)*	0.834 (0.426)*	0.441 (0.379)	0.584 (0.313)*	0.657 (0.305)*	−0.680 (0.779)	−0.448 (0.764)	−0.304 (0.808)	−0.033 (0.786)
Female without male support	−0.364 (0.394)	−1.146 (0.507)*	−0.043 (0.214)	0.031 (0.262)	−0.406 (0.192)*	−0.076 (0.220)	−0.293 (0.141)*	0.094 (0.177)	0.029 (0.351)	0.802 (0.444)	−0.025 (0.364)	0.910 (0.456)*
**Household head education level**												
No schooling	Reference	Reference	Reference	Reference	Reference	Reference	Reference	Reference	Reference	Reference	Reference	Reference
Primary school completed	0.593 (0.545)	0.477 (0.613)	0.868 (0.263)**	0.579 (0.274)*	0.691 (0.236)**	0.330 (0.230)	0.478 (0.172)**	0.282 (0.185)	0.232 (0.431)	−0.112 (0.463)	0.534 (0.445)	0.159 (0.476)
Above primary school	0.238 (0.644)	0.056 (0.613)	0.850 (0.319)**	0.294 (0.350)	1.008 (0.286)***	0.281 (0.295)	0.967 (0.209)***	0.418 (0.237)	1.302 (0.522)*	−0.121 (0.594)	1.851 (0.539)**	0.308 (0.611)
**Currently growing sweetpotato**												
No	Reference	Reference	Reference	Reference	Reference	Reference	Reference	Reference	Reference	Reference	Reference	Reference
Yes	−0.280 (0.442)	−0.397 (0.445)	0.748 (0.150)***	0.463 (0.150)**	1.566 (0.128)***	1.153 (0.126)***	0.511 (0.099)***	0.344 (0.101)**	0.684 (0.248)**	0.420 (0.254)	0.730 (0.257)**	0.412 (0.261)
**Agricultural activity**												
Principal	Reference	Reference	Reference	Reference	Reference	Reference	Reference	Reference	Reference	Reference	Reference	Reference
Secondary	0.113 (0.442)	0.015 (0.478)	0.046 (0.248)	−0.177 (0.258)	0.208 (0.220)	−0.210 (0.214)	0.284 (0.161)	−0.230 (0.172)	2.070 (0.396)***	1.303 (0.432)**	2.344 (0.409)***	1.512 (0.444)**
**Sold agricultural /livestock products in past 2 years, yes**												
No	Reference	Reference	Reference	Reference	Reference	Reference	Reference	Reference	Reference	Reference	Reference	Reference
Yes	0.122 (0.274)	0.305 (0.287)	−0.318 (0.145)*	−0.299 (0.145)*	0.052 (0.131)	−0.193 (0.122)	0.344 (0.095)**	0.097 (0.098)	0.742 (0.237)*	0.152 (0.246)	0.876 (0.245)**	0.203 (0.253)
**Casual labor in past 2 years**												
No	Reference	Reference	Reference	Reference	Reference	Reference	Reference	Reference	Reference	Reference	Reference	Reference
Yes	−0.284 (0.268)	−0.132 (0.267)	−0.222 (0.147)	−0.047 (0.141)	−0.132 (0.133)	0.060 (0.119)	−0.072 (0.098)	0.049 (0.095)	−0.352 (0.242)	−0.083 (0.239)	−0.352 (0.251)	−0.058 (0.246)
**Involved in informal business with past 2 years**												
No	Reference	Reference	Reference	Reference	Reference	Reference	Reference	Reference	Reference	Reference	Reference	Reference
Yes	−0.918 (0.354)**	−1.042 (0.353)**	−0.139 (0.185)	−0.083 (0.178)	0.132 (0.166)	0.030 (0.149)	0.231 (0.122)*	0.075 (0.120)	0.977 (0.302)**	0.569 (0.300)*	1.126 (0.312)**	0.660 (0.309)*
**Salaried employment within past 2 years**												
No	Reference	Reference	Reference	Reference	Reference	Reference	Reference	Reference	Reference	Reference	Reference	Reference
Yes	−0.150 (0.476)	0.039 (0.544)	0.440 (0.271)	0.419 (0.294)	0.562 (0.243)*	0.268 (0.248)	0.641 (0.178)**	0.268 (0.199)	1.729 (0.441)***	0.340 (0.499)	1.736 (0.457)***	0.030 (0.513)
**Self-employment within past 2 years**												
No	Reference	Reference	Reference	Reference	Reference	Reference	Reference	Reference	Reference	Reference	Reference	Reference
Yes	0.534 (0.256)*	0.570 (0.274)*	0.865 (0.151)***	0.545 (0.156)**	0.967 (0.135)***	0.382 (0.131)**	0.412 (0.101)***	0.072 (0.106)	0.888 (0.250)**	0.188 (0.264)	1.060 (0.259)***	0.235 (0.272)
Wealth index of household	0.043 (0.035)	0.062 (0.042)	0.008 (0.017)	−0.021 (0.022)	0.102 (0.015)***	0.057 (0.018)**	0.099 (0.011)***	0.083 (0.015)***	0.229 (0.028)***	0.163 (0.037)***	0.258 (0.029)***	0.187 (0.038)***
**Source of consumed OFSP**												
Market purchase	Reference	Reference	Reference	Reference	Reference	Reference	Reference	Reference	Reference	Reference	Reference	Reference
Own field	−0.168 (0.337)	−0.234 (0.347)	0.104 (0.281)	0.186 (0.224)	0.070 (0.245)	0.384 (0.241)	0.147 (0.207)	0.316 (0.201)	−0.396 (0.515)	0.179 (0.486)	−0.231 (0.529)	0.386 (0.495)

*^§^Unadjusted model signifies the bivariate association between dependent and independent variables alone, whereas adjusted analysis is a reduced model, taking into account all the covariates that showed a significant association*.

*** p-value significant at a two-sided alpha of <0.0001;

**p-value significant at a two-sided alpha of <0.01;

*p-value significant at a two-sided alpha of <0.05.

## Discussion

This study evaluated the impact of nutrition social behavior change on caregiver nutrition KPs in rural Tanzania. Among caregivers who had attended at least one nutrition group meeting where nutrition and health talks were provided, health and childcare knowledge, household and young child dietary diversity, and VA intake were significantly greater compared with their non-participating counterparts. Similar findings were observed among women attending antenatal care (ANC) in rural western Kenya where women who had attended the ANC clinic at least one time had significantly better health knowledge scores and received malaria or anthelmintic treatment compared with their non-attending counterparts (29). In our study, we found that attendance at nutrition group meetings improved diet quality for both household members and children under 5 years of age. There was also an improvement in dietary VA intake among household members and young children. This parallels findings from a study in Bangladesh that used a multi-wave cohort design to evaluate a nutrition intervention program with the use of BCC strategies to improve infant feeding practices and growth indicators in the first 2 years of life (8). The Bangladeshi study observed an improvement in the rates of complementary feeding initiation and diet quality of infants at 9 months of age among women who received nutrition education compared to non-recipients. In our study, we observed a greater magnitude of the associations between participation and health/nutrition KPs, where, for caregivers who attended four or more nutrition meetings, the likelihood of having improved KPs (HKS, dietary diversity, and VA intakes) was at least one-and-half times greater compared with those who attended three or fewer meetings. This is an indication that health and nutrition education provided at these nutrition group meetings potentially confers substantive improvements in health and childcare knowledge as well as improved practices, leading to improved dietary diversity and dietary VA intakes for young children and household members.

In our study, we did not observe significant differences in nutrition knowledge outcomes by participation in nutrition group meetings, and this might potentially be a result of the presence of community nutrition committees in some of the villages apart from the project-implemented nutrition group meetings. This might have resulted in homogeneity in caregiver KPs irrespective of participation in nutrition group meetings implemented by our project and led by the CHWs. Other potential reasons for this lack of difference in caregiver nutrition knowledge might be due to reduced uptake of the information offered at the nutrition meetings or ineffective nutrition messaging and counseling at the meetings (29, 31). Cooking demonstrations of various recipes for nutritious diets formed an integral part of the nutrition group meetings. This might have explained the improved dietary diversity and VA intake among participants, independent of the observed null impact observed for nutrition knowledge among participants and non-participants. Women who were present at the nutrition meetings benefitted from direct involvement in recipe preparation of nutritious diets for improved young child complementary feeding and household diets, even when they demonstrated reduced knowledge of the theoretical teachings on nutrition implemented by the trained CHWs.

In addition to attendance at nutrition group meetings, the caregiver education level was positively associated with health and childcare knowledge, dietary diversity, and VA intakes. A higher maternal education level has positively been associated with health and nutrition knowledge and dietary diversity among mothers attending ANC clinics in western Kenya (29) and with improved maternal health knowledge scores in Turkey, Laos, and China (32–34). Additionally, we observed that the cultivation of sweetpotato by households was associated with improved caregiver health and childcare knowledge, dietary diversity, and VA intake among household members and young children. This was similar to our previous observations in rural western Kenya that linked the promotion and distribution of biofortified OFSP to health services and enhanced nutrition for pregnant and lactating women, improved maternal nutrition and healthcare knowledge, and increased OFSP and VA intake (35). That intervention also reduced the odds of anemia and VAD, indicated as low retinol-binding protein, at late prenatal and at 9-month postpartum, respectively.

In the current study, although participation in nutrition group meetings was associated with a significant improvement in knowledge and improved diet quality as well as increased consumption of VA-rich foods, we did not observe a positive association between nutrition behavior change exposure and increased consumption of OFSP. Similar reports have indicated that interventions aimed at change in dietary practices require extended exposure before significant changes in practices are observed (35–38). In spite of 89% of participants of nutrition group meeting reporting to have cultivated OFSP, only 49% of them reported the consumption of OFSP within the past 24 h. Given that the harvesting season for sweetpotato in the project intervention areas was late April to late May, this low consumption was expected because the survey took place in August/September, 3 months after the harvesting season. However, this limited association testifies to the difficulties in achieving nutrition SBCC in addition to potential intervention limitations associated with project implementation such as low uptake of community-based activities such as nutrition group meetings (35, 39). Our study also reported a positive association between self-employment and nutrition knowledge, health and childcare knowledge, and household DDS. Similarly, there was a positive impact of household wealth on household and young child diet quality as well as on dietary VA intakes. This indicates that economically stable households have the means to access nutritious foods, including VA-rich foods, for household consumption. Although evidence exists that caregiver employment presents with maternal “double burden” of increased demands for labor and economic activity to the detriment of adequate childcare responsibilities (40), the positive association between maternal employment and improved KPs found in our study was in direct contrast. Women who were involved in the income-generating activities might have had the means and autonomy to decide on the acquisition of a quality diet for the family and children in our intervention sites.

The strengths of our study include the training, refresher-training, and periodic monitoring of the CHWs who were the implementers of the community-based nutrition group meetings. This ensured quality messages and education were being provided to the participating caregivers. However, there are some limitations to our intervention. The development of outcome variables for caregiver KPs on infant feeding practices was based on maternal recall and not from direct observation of feeding practices. Further, the nutrition SBCC messages used in the project's intervention may have resulted in desirability-biased responses (8). Although the DDS is considered to be a valid proxy indicator of nutrition adequacy and is often employed in resource-poor settings (37), the use of the 24-h recall to construct the DDS does not account for within-individual variability (41).

In conclusion, this study showed that caregiver participation in nutrition SBCC was positively associated with improved health and childcare knowledge, household and young child dietary diversity, and VA intake compared with non-participants. The magnitude of the association was greater for caregivers who attended at least four meetings. Our findings emphasize the need for programs that seek to address the issues present in the use of nutrition SBCC at the community level to improve maternal or caregiver KPs and subsequently to improve the nutrition status of infants and young children. There is the need to strengthen the nutrition content of such training models and implement periodic monitoring of the activities of the implementers of the nutrition SBCC at the community level to ensure that messages being delivered to the participating caregivers are of the required quality. Finally, qualitative research is needed in this context to provide an in-depth understanding of the determinants of health and nutrition KPs among caregivers of infants and young children.

## Data Availability Statement

The datasets presented in this study can be found in online repositories. The names of the repository/repositories and accession number(s) can be found in the article/Supplementary Material.

## Ethics Statement

The studies involving human participants were reviewed and approved by the Commission for Science and Technology/ COSTECH, Tanzania. Written informed consent was obtained from all subjects. The patients/participants provided their written informed consent to participate in this study.

## Author Contributions

FG wrote the manuscript and had primary responsibility for the final content of the manuscript and analyzed the data. FG and RA-A designed the analysis approach and designed the research. FG, HO, and RK conducted the research. All authors read and approved the final manuscript.

## Funding

Funding support for this work was provided by USAID (MTO 069018) and UKAID (Programme code: 204022).

## Conflict of Interest

The authors declare that the research was conducted in the absence of any commercial or financial relationships that could be construed as a potential conflict of interest.

## Publisher's Note

All claims expressed in this article are solely those of the authors and do not necessarily represent those of their affiliated organizations, or those of the publisher, the editors and the reviewers. Any product that may be evaluated in this article, or claim that may be made by its manufacturer, is not guaranteed or endorsed by the publisher.
