# Supplementary material for: Association Between Nutrition Social Behavior Change Communication and Improved Caregiver Health and Nutrition Knowledge and Practices in Rural Tanzania
Source: Front Public Health. 2022 Jun 20;10:736666. doi: 10.3389/fpubh.2022.736666 (PMC9251192; doi:10.3389/fpubh.2022.736666)
Supplement: Supplementary file 1 [file Table_1.pdf]

Annex 1

Table A1: Survey items used to generate dependent summative scores of nutrition knowledge, health and healthcare knowledge, attitudes, and dietary diversity

Outcomes of Interest	Dependent Variables	Survey items	Variable Construction
Knowledge Scores	Nutrition Knowledge Score	• Knowledge of three food groups<br>• Main sources of energy<br>• Main source of body-building foods<br>• Main sources of protective foods	Equally-weighted sub-scales per question, and summative score
	Health and healthcare Knowledge Score	• Eating well (good quality foods).<br>• Avoid drinking alcohol<br>• Do not Smoke<br>• Attend ANC-care clinic<br>• Take iron tables or iron syrup<br>• Protect from malaria with nets/drugs<br>• Check HIV Status<br>• Other (Avoid stress/exercise) (equally-weighted sub-scale)<br>• When should a pregnant woman start going to ANC clinic?<br>• How many times to attend ANC<br>• List 3 activities that occur at ANC clinic (equally-weighted sub-scale)	Summative score
Practices	Dietary Diversity	Household food consumption for 12 groups of foods (1 item per food group type)	Summative score
	Food intake	Eating less, more or the same amount of food during pregnancy	N/A
	Seeking treatment	Taken drugs to prevent malaria and intestinal worms during pregnancy	N/A
